# Supplementary material for: COZOID: contact zone identifier for visual analysis of protein-protein interactions
Source: BMC Bioinformatics. 2018 Apr 6;19:125. doi: 10.1186/s12859-018-2113-6 (PMC5889581; doi:10.1186/s12859-018-2113-6)
Supplement: Supplementary file 2 — Software build. Executable binary file of the software tool. (ZIP 118,784 kb) [file 12859_2018_2113_MOESM2_ESM.zip › cozoid/platform/docs/testng-6.5.1-javadoc.zip!/org/testng/SuiteRunState.html]

SuiteRunState (TestNG)


---


|  |  |  |
| --- | --- | --- |
| **Overview** \| **Package** \| **Class** \| **Tree** \| **Deprecated** \| **Index** \| **Help** | |  |
| **PREV CLASS**   **NEXT CLASS** | **FRAMES**    **NO FRAMES**     **All Classes** |  |
| SUMMARY: NESTED \| FIELD \| CONSTR \| METHOD | DETAIL: FIELD \| CONSTR \| METHOD |  |


---


## org.testng Class SuiteRunState

```
java.lang.Object
  org.testng.SuiteRunState
```

**All Implemented Interfaces:**: java.io.Serializable

---

``` public class SuiteRunState extends java.lang.Object implements java.io.Serializable ```

A state object that records the status of the suite run. Mainly used to
figure out if there are any @BeforeSuite failures.

**Author:**
:   Alexandru Popescu

**See Also:**: Serialized Form

---

**Constructor Summary**

`SuiteRunState()`


| **Method Summary** | |
| --- | --- |
| `void` | `failed()` |
| `boolean` | `isFailed()` |

**Methods inherited from class java.lang.Object**

`clone, equals, finalize, getClass, hashCode, notify, notifyAll, toString, wait, wait, wait`

**Constructor Detail**

### SuiteRunState

```
public SuiteRunState()
```


**Method Detail**

### failed

```
public void failed()
```

---


### isFailed

```
public boolean isFailed()
```


---


|  |  |  |
| --- | --- | --- |
| **Overview** \| **Package** \| **Class** \| **Tree** \| **Deprecated** \| **Index** \| **Help** | |  |
| **PREV CLASS**   **NEXT CLASS** | **FRAMES**    **NO FRAMES**     **All Classes** |  |
| SUMMARY: NESTED \| FIELD \| CONSTR \| METHOD | DETAIL: FIELD \| CONSTR \| METHOD |  |


---
